# Genomic and biological insights of bacteriophages JNUWH1 and JNUWD in the arms race against bacterial resistance

**DOI:** 10.3389/fmicb.2024.1407039

**Published:** 2024-06-26

**Authors:** Hengwei Zhang, Jiajia You, Xuewei Pan, Yanglu Hu, Zan Zhang, Xian Zhang, Weiguo Zhang, Zhiming Rao

**Affiliations:** ^1^Key Laboratory of Industrial Biotechnology of the Ministry of Education, School of Biotechnology, Jiangnan University, Wuxi, China; ^2^National Engineering Research Center of Cereal Fermentation and Food Biomanufacturing, Jiangnan University, Wuxi, China

**Keywords:** bacteriophages, genomic analysis, *Escherichia coli*, Siphoviridae, LPS, lptD

## Abstract

The coevolution of bacteria and bacteriophages has created a great diversity of mechanisms by which bacteria fight phage infection, and an equivalent diversity of mechanisms by which phages subvert bacterial immunity. Effective and continuous evolution by phages is necessary to deal with coevolving bacteria. In this study, to better understand the connection between phage genes and host range, we examine the isolation and genomic characterization of two bacteriophages, JNUWH1 and JNUWD, capable of infecting *Escherichia coli*. Sourced from factory fermentation pollutants, these phages were classified within the *Siphoviridae* family through TEM and comparative genomic analysis. Notably, the phages exhibited a viral burst size of 500 and 1,000 PFU/cell, with latent periods of 15 and 20 min, respectively. They displayed stability over a pH range of 5 to 10, with optimal activity at 37°C. The complete genomes of JNUWH1 and JNUWD were 44,785 bp and 43,818 bp, respectively. Phylogenetic analysis revealed their close genetic relationship to each other. Antibacterial assays demonstrated the phages’ ability to inhibit *E. coli* growth for up to 24 h. Finally, through laboratory-driven adaptive evolution, we successfully identified strains for both JNUWH1 and JNUWD with mutations in receptors specifically targeting lipopolysaccharides (LPS) and the *lptD* gene. Overall, these phages hold promise as additives in fermentation products to counter *E. coli*, offering potential solutions in the context of evolving bacterial resistance.

## 1 Introduction

Bacteriophages represent an intriguing avenue in the ongoing battle to control bacterial infections. These viruses, which specifically target bacteria, have long been recognized for their potential in combatting bacterial infections (Chang et al., 2022; Sanmukh et al., 2023). Since their discovery in the early 20th century, phages have garnered increasing attention for their unique ability to infect and lyse bacterial cells (Chen et al., 2023). This natural phenomenon has been the driving force behind endeavors to harness phages as potential therapeutic agents against bacterial pathogens (Sharma et al., 2017). The dynamic co-evolution between bacteria and phages has not only shaped the intricate resistance systems of bacteria but has also propelled the adaptation and diversification of phages (Koonjan et al., 2020; Venturini et al., 2022).

This study delves into the isolation and analysis of two bacteriophages, JNUWH1 and JNUWD, with the capability to infect *E. coli*. Phages isolated from natural environments often demonstrate low burst sizes and weak inhibitory effects, and they tend to have limited application potential (Chevallereau et al., 2021). However, in fermentation systems, extremely low Multiplicity of Infection (MOI, MOI < 0.001) can lead to system collapse. These two bacteriophages were isolated from collapsed fermentation medium. They exhibit short replication cycles and high burst volume of cleavage, as well as genetic stability in the infection cycle. Therefore, research directed toward these phages holds significant relevance (Tabib-Salazar et al., 2017; Chen et al., 2023; Wei et al., 2023; Xiao et al., 2023; Zhang et al., 2023). Our research aims to illuminate the genetic makeup and taxonomic classification of these phages. Through this exploration, we seek to not only contribute to the broader understanding of phage diversity but also to highlight their potential applications (Wei et al., 2023).

This study stands at the intersection of historical phage research and contemporary applications. Studying phages’ biological and receptors characteristics can help us understand the life process of phages and help solve the huge economic losses caused by phage infection in the fermentation process. In addition, through this work, we strive to contribute to a growing body of knowledge that also has the potential to revolutionize approaches to bacterial control and treatment (Duan et al., 2022), even though the phages in this study have not yet been found to lyse pathogenic microorganisms.

## 2 Materials and methods

### 2.1 Phage isolation and purification

The samples were obtained from two synthetic biology products factories in China’s Jiangsu and Hubei provinces that specialize in industrial microbes. The fermentation broth, potentially harboring bacteriophages, underwent centrifugation (10 min at 12,000 g, 4°C) and filtration (through a 0.22 μm syringe filter, Nylon) for enrichment. Subsequently, 300 μL of *E. coli* BL21 from a 5-h culture and 100 μL of the fermented liquid after filtration were combined in 10 mL LB broth, followed by an overnight incubation at 37°C with agitation (180 rpm). Subsequent validation of phage presence was accomplished through continuous monitoring of OD_600_ values during the growth of *E. coli*. The bacteriophage was purified using a double-layer agar method, wherein 100 μL of the bacteriophage suspension, diluted to 10^3^ pfu/ml, was mixed with 300 μL of host bacterial suspension in 5 mL of semi-solid LB medium (containing agar powder at a concentration of 0.5%). This mixture was then poured into a solid LB medium (containing agar powder at a concentration of 1%) and allowed to incubate overnight at 37°C. Following this, a singular plaque containing a purified phage was selected. Individual plaques were carefully picked and introduced into *E. coli* medium for the purpose of amplification. The resulting lysate underwent a series of centrifugation and filtration processes, culminating in its preservation in buffer at 4°C to ensure optimal stability for subsequent experiments (Luong et al., 2020).

The phages JNUWH1 and JNUWD were subjected to purification for TEM analysis and purified phage lysates were stained with 1% (w/v) uranyl acetate for 2 min prior to examination using a transmission electron microscope (Hitachi H-7650) (Wei et al., 2023).

### 2.2 Host range determination of phage JNUWH1 and JNUWD

The bacteriostatic effect of phages was assessed against 15 colistin-resistant *E. coli* strains using an OD-based turbidimetric method (OD_600_) (Xiao et al., 2023). The cultures were treated with 20 μL of purified phages at 10^7^ PFU/mL, and the control group contained only bacteria. A control (bacteria only) and experimental (bacteria with phage) group were set up for each bacterial strain tested and incubated in a petri dish at 37°C for 8 h, and OD_600_ readings were taken every 30 min to determine whether the bacteria were lysed by comparing the changes of OD_600_ in the experimental group compared to the control group. In addition, for the strains whose growth was inhibited, host range was further verified by observing whether plaques formed after dropping bacteriophages on the double-layer plate of *E. coli* (Chen et al., 2023).

### 2.3 One-step growth curve of phage JNUWH1 and JNUWD

The one-step growth experiment was carried out according to established protocol with minor modifications (Koonjan et al., 2020). The host strain *E. coli* BL21 was grown to an early logarithmic stage (OD600 = 0.45–0.55) and diluted with LB broth to 10^6^ CFU/mL. The diluted bacteriophage was mixed with the bacterial culture at MOI 1, incubated at 37°C, and samples were taken every 5 min, and then centrifuged. The supernatant was diluted to an appropriate concentration, and the host *E. coli* was mixed with phage through a double-layer plate and cultured at 37°C for 6 h. The phage titer was determined by measuring the number of single plaques.

### 2.4 Biological characteristics of phage JNUWH1 and JNUWD

For thermal stability testing, the method of Chen et al. (2023) was used where phages (10^6^ PFU/mL) were incubated at different temperatures for 60 min. For pH stability, phages (10^7^ PFU/mL) were exposed to varying pH (2–12) at 4°C for 60 min. Phage titers were measured at 60 min using the double-layer agar method.

To calculate the adsorption rate, the strain *E. coli* to be detected in this study was cultured until reaching the early logarithmic growth phase (OD600 = 0.45–0.55). Subsequently, the bacteriophage was combined with the bacterial culture at a multiplicity of infection (MOI) of 1, followed by incubation at 37°C. The control group (S_0_) underwent the same procedure using LB medium without bacteria. Samples were collected after 15 min and subsequently centrifuged. The resulting supernatant was further diluted to an appropriate concentration. *E. coli* BL21 was then mixed with the bacteriophage using a double-layer plate method and incubated at 37°C for 6 h. The phage titer (S) was determined by quantifying the number of single plaques formed. The calculation method for bacteriophage adsorption rate was defined as “1 - S/S_0_”.

### 2.5 Genome sequencing and bioinformatic analysis

For next-generation sequencing library preparation, phage genomic DNA (200 μg) was precisely fragmented using Covaris S220 (Duty Factor 10%, Peak/Displayed Power 175 W, Cycles/Burst 200, Duration 50 s, Temperature 4–8°C), resulting in fragments averaging 300-350 bp. Following this, the End Prep Enzyme Mix (#ND608, Vazyme, Nanjing, China) was applied for accurate end-repair, 5′ phosphorylation, and 3′ adenylation, enabling adaptor attachment at both ends. The adaptor-ligated DNA underwent precise size selection with DNA Cleanup beads (#N411, Vazyme, Nanjing, China). Subsequently, each sample underwent 8 cycles of PCR amplification, designed for flowcell annealing, with a primer featuring a six-base index for multiplexing. The PCR products were then thoroughly purified and validated using an Agilent 2,100 Bioanalyzer. The qualified libraries were subjected to pair-end (PE150) sequencing on the Illumina Novaseq 6,000 System. The reads underwent rigorous quality control, followed by assembly using Velvet, with gap-filling conducted through SSPACE (version 3.0) and GapFiller (version 1–10) (Zerbino and Birney, 2008; Zerbino et al., 2009; Boetzer et al., 2011; Boetzer and Pirovano, 2012; Hunt et al., 2014). Gene identification used the software program Prodigal (version 3.02) (Stanke et al., 2006; Tillich et al., 2017), while tRNAs were detected via tRNAscan-SE2.0 with default settings, and other RNA types were identified using Rfam (version 1.1.2). Coding genes were annotated through BLAST against the NCBI nr database, followed by functional annotation using the Gene Ontology (GO) database, and pathway annotation with the Kyoto Encyclopedia of Genes and Genomes (KEGG) database (Ogata et al., 1999; Harris et al., 2004). The alignment between two genomes was conducted using the nucmer algorithm with specific parameters (−-maxgap = 200, −-mincluster = 50), ensuring a maximum gap of 200 base pairs and a minimum of 50 matches per cluster, subsequent data refinement, employing delta-filter with stringent criteria (60% minimum similarity, 500 bp minimum match length), yielded a refined delta file (Kurtz et al., 2004).

vContact2 v0.11.3 has been used to predict species relationships of phages (Jang et al., 2019)， and PhageTerm v4.1 software was used to determine the genome ends (Garneau et al., 2017). The evolutionary relationship between the two phages within was investigated by performing a BLASTn^1^ search against the nr database for the tail fiber protein and terminase large subunit. Identified homologous sequences were then subjected to phylogenetic analysis. Utilizing the Maximum Likelihood method based on the Tamura and Nei (1993), the evolutionary history was inferred. For robust results, we initiated the heuristic search by automatically generating initial trees. This was accomplished by employing both the Neighbor-Join and BioNJ algorithms on a pairwise distance matrix, which was estimated using the Maximum Composite Likelihood (MCL) approach. The topology with the highest log likelihood value was selected. Positions with less than 95% site coverage were excluded, allowing for fewer than 5% alignment gaps, missing data, and ambiguous bases at any position. These evolutionary analyses were conducted using MEGA7 (Kumar et al., 2016). Unless otherwise specified, default parameters were used.

### 2.6 Laboratory adaptive evolution

By drawing on previous studies using laboratory adaptation, bacteria with phage resistance were evolved by mixing bacteriophages with their host at MOI 1 and cultured them until the OD_600_ drops, which means that the bacteria were lysed. After the lysate was further cultured for 24 h at 37°C, the single colonies were separated by streaking and the phage resistance was verified by double-plate method， as described in section 2.1 (Huang et al., 2022). To validate the phage-resistant bacterial strains, they were initially cultured in 10 mL of LB liquid medium at 37°C for 8 h. Subsequently, the cultures were transferred to 50 mL of LB liquid medium and incubated overnight under the same conditions. After incubation, the cultures were centrifuged at 8000 rpm for 10 min, and the supernatant was discarded. The resulting bacterial pellet was flash-frozen in liquid nitrogen and sent to GENEWIZ (Suzhou China) for resequencing. The resequencing was conducted according to the protocols specified by the company.

### 2.7 Strain and plasmid construction

In the process of evolution, we found that some candidate genes that may affect the interaction between bacteriophages and bacteria may stop the phage adsorption process by knockout, and supplementing these knockout genes by plasmids can further prove the necessity of these genes in the phage adsorption process. The method used for gene knockout and integration involved the CRISPR-Cas9 system (Li et al., 2021). Regarding plasmid construction, the amplified target gene sequence and the plasmid vector were each digested with Bam*HI* (Takara, Dalian, China) and Hin*dIII* (Takara, Dalian, China) enzymes separately. Following digestion, the linearized plasmid vector and the target gene fragment were ligated together using T4 DNA ligase (Vazyme, Nanjing, China), facilitating the integration of the target gene into the plasmid vector. The constructed plasmid was introduced into corresponding *E. coli* by chemical transformation method.

## 3 Result

### 3.1 Morphological features of JNUWH1 and JNUWD

**Figure 1:**
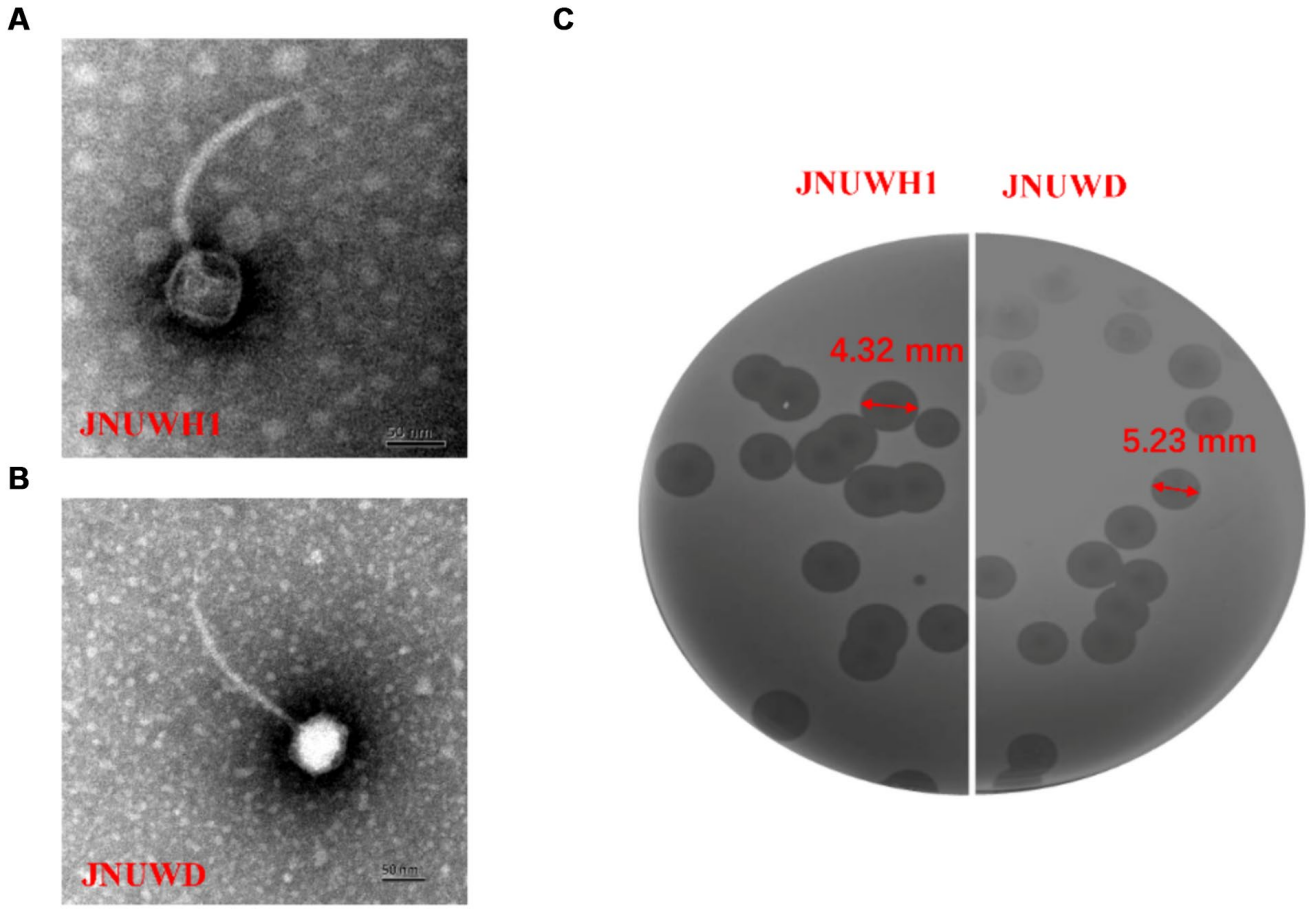
*Escherichia* phage JNUWH1 and JNUWD. **(A)** Transmission electron micrograph of phage JNUWH1; **(B)** transmission electron micrograph of phage JNUWD; **(C)** plaques formed on the lawn of *E. coli* BL21.

Bacteriophage JNUWD was isolated and purified from fermentation vessels at a biotechnology company in Jiangsu China that employs *E. coli* as the microbial host for production. The phage JNUWH1 was obtained from a contaminated fermentation vessel in an academic laboratory environment different from the one where JNUWD was found. The two phages had been repeatedly responsible for fermentation failures due to persistent contamination. Transmission Electron Microscopy (TEM) analysis confirmed the classification of both JNUWD and JNUWH1 as long-tailed lytic bacteriophages. Phage JNUWH1 has a 70 nm diameter head and a 185 nm long tail (Figure 1A) and phage JNUWD has a 50 nm diameter head and a 160 nm long tail (Figure 1B). The two phages exhibit similar bull-eyed plaque morphologies characterized, indicating a high lytic potential. Based on the turbidity of the plaque, both phages have bull-eyed plaques, and these represent depolymerase activity, representing the importance in biofilm removal (Sanmukh et al., 2023; Figure 1C).

### 3.2 Biological characteristics of phage JNUWH1 and JNUWD

**Figure 2:**
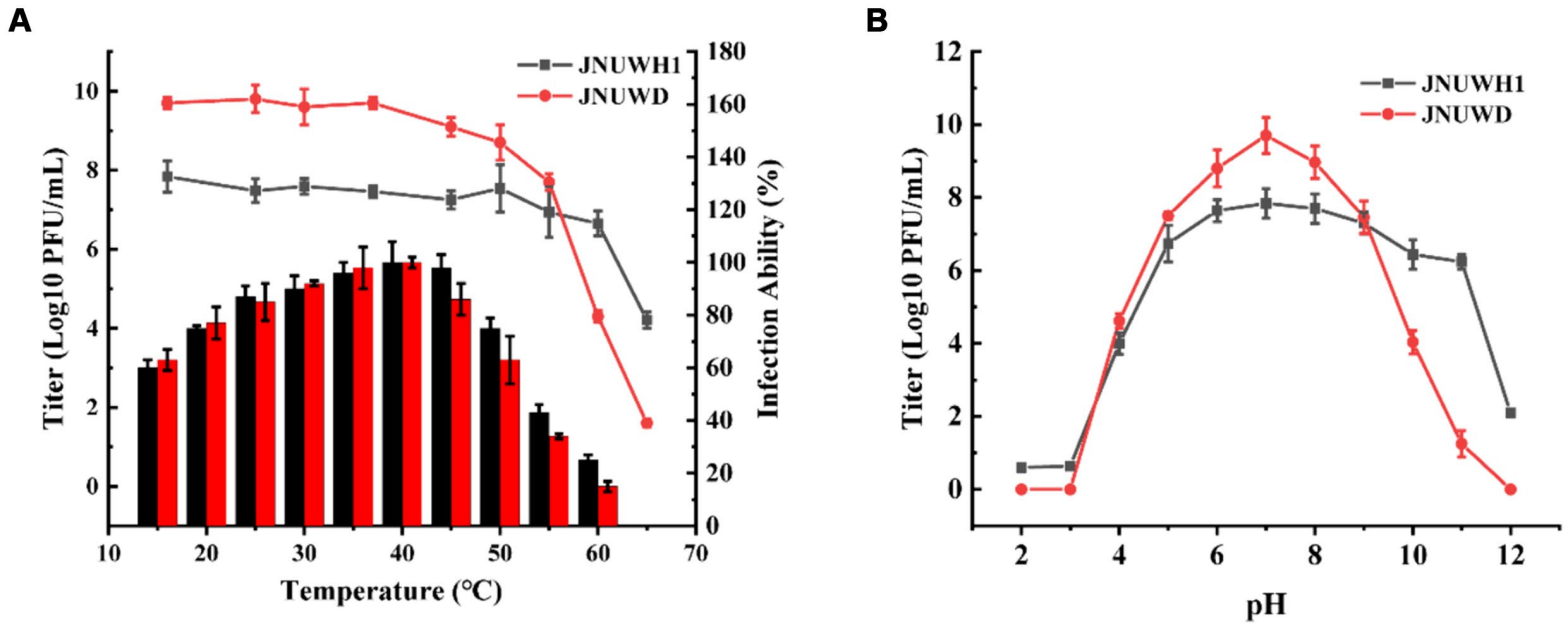
Biological characteristics of phage JNUWH1 and JNUWD. **(A)** Thermostability and Optimal infection temperature of phage JNUWH1 and JNUWD, the line graph represents the temperature stability, and the bar graph represents the optimal infection temperature. **(B)** pH stability of JNUWH1 and JNUWD. The error bars indicate the standard deviation. The result was calculated based on three repeated experiments.

In assessing the thermal stability of the two bacteriophages, JNUWH1 demonstrated notable stability at temperatures below 60°C, while for JNUWD, temperatures below 50°C within a one-hour timeframe had a limited impact on phage stability (Figure 2A). Optimal infection temperatures for both bacteriophages were observed at 40°C (Figure 2A). This may be explained by the heightened activity of the host bacteria at this temperature, which provides an optimal environment for the bacteriophage life cycle to progress. In terms of pH sensitivity, both bacteriophages exhibited low stability when subjected to incubation in different buffering solutions for 1 h, particularly at pH levels below 5. Notably, there was a significant reduction in JNUWH1 infectivity observed at pH levels exceeding 11, whereas JNUWD exhibited instability at pH levels as high as 10 (Figure 2B). This disparity may be attributed to the differential pH stability of capsid proteins and receptor-binding proteins associated with the tail fibers, influencing the interaction dynamics of the bacteriophages (Leprince and Mahillon, 2023; Supplementary Figure S1).

### 3.3 One step curve and lysis spectrum of phages

**Figure 3:**
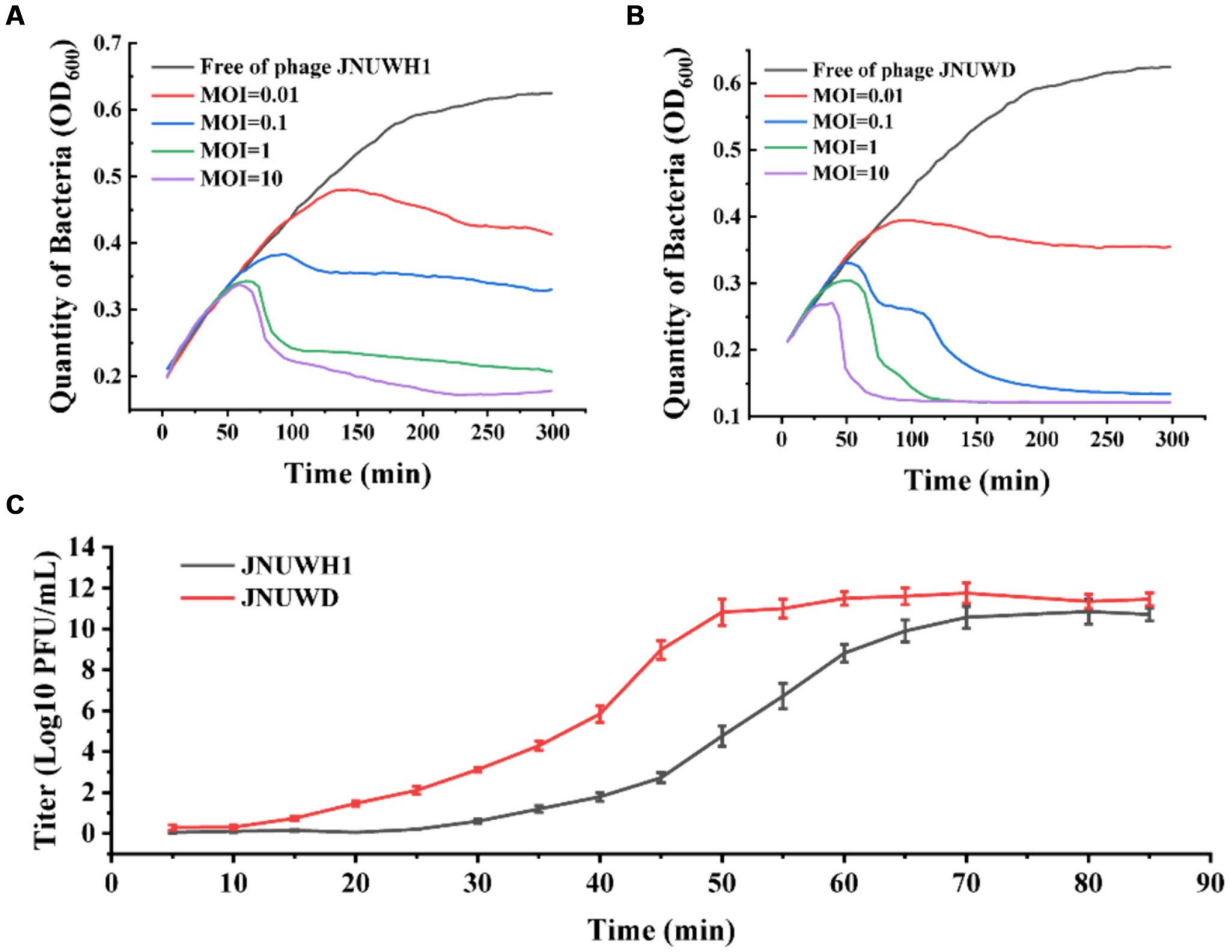
The infection curve and the one-step growth curves of JNUWH1 and JNUWD. **(A)** The horizontal axis represents the coincubation time of JNUWH1 at different MOIs. **(B)** The horizontal axis represents the coincubation time of JNUWD at different MOIs **(C)** One step growth curves of JNUWH1 and JNUWD in the presence of the *E. coli* BL21 host. The error bars indicate standard deviation. The result was calculated based on three repeated experiments.

It was observed that both bacteriophages exhibited efficacy across a spectrum of MOI levels (Figures 3A,B). Under MOIs of 0.01, 0.1, 1.0, and 10, the growth of *E. coli* BL21 gradually declined within a timeframe of 40–60 min. After 24 h there was no proliferation of the host bacteria in the cultures. The one-step growth curves of *E. coli* indicate that the latent periods for JNUWH1 and JNUWD are 20 and 15 min, respectively, with corresponding burst periods of 55 and 40 min and the viral burst size of the phages were 500 and 1,000 PFU/cell. The final titers of both bacteriophages exceeded 10^9^ pfu/mL (Figure 3C). These data underscore the potent inhibitory capacity of these two virulent bacteriophages toward *E. coli* growth. Consequently, in industrial fermentation processes, proactive measures are imperative to prevent and mitigate the impact of such bacteriophages.

We evaluated the host range of the two bacteriophages against fifteen strains of *E. coli* from diverse sources. Measurements of OD_600_ indicated that bacteriophage JNUWH1 and JNUWD demonstrated inhibitory activity only against three specific strains of *E. coli* (Table 1). The two types of bacteriophages lacked infectivity against the tested pathogenic *E. coli* (Supplementary Table S1).

**Table 1:** The host range of JNUWH1 and JNUWD.

Specific name	Serotypes	Source of strains	Infection of JNUWH1	Infection of JNUWD
*E. coli*	O157:H7	ATCC 43895		
*E. coli*	O55:H7	CB9615	+++	+++
*E. coli*	O157:H7	ATCC 35150		
*E. coli*	O157:H7	ATCC 700728		
*E. coli*	O111:K58:H21	ATCC 29552		
*E. coli*	O103:H8	ATCC 11229		
*E. coli*	O78:K80	CICC 10421		
*E. coli*	O78:H11	ATCC 35401		
*E. coli*	O128:H7	CDC 1458–80		
*E. coli*	O114:H2	ATCC 23540		
*E. coli*	O145:H2	BAA-2585		
*E. coli*	O26:H11	ATCC 2003–3,014		
*E. coli*		ATCC 10798	+++	+++
*E. coli*		CCTCC/B2020	+++	+++
*E. coli*	O111:H8	CDC 1997–3,215		

+++: Represents the sensitivity of the strain to phage.

### 3.4 Genomic characteristics and taxonomy

**Figure 4:**
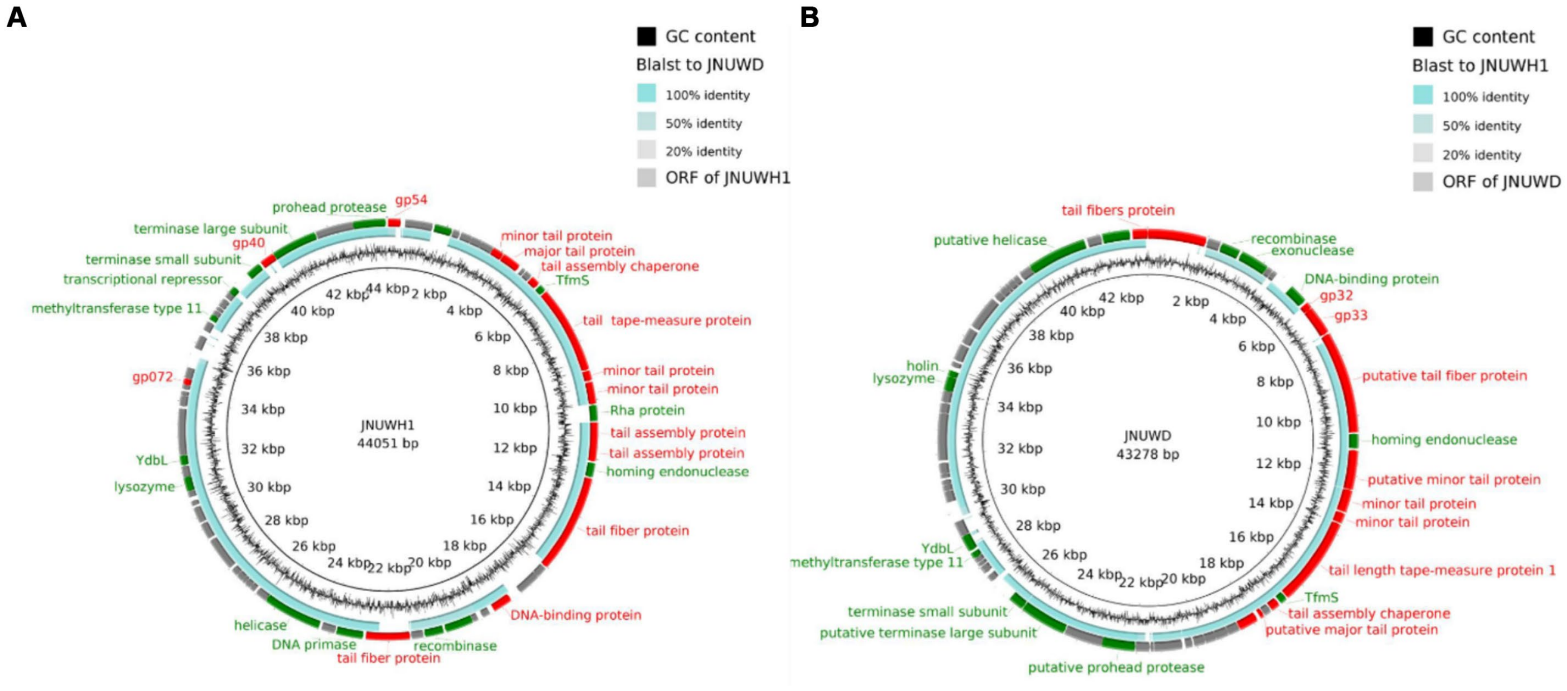
**(A)** Genome map and functional protein identity of phage JNUWH1. **(B)** Genome map and functional protein identity of phage JNUWD. The arrows indicate the direction of transcription of each gene. Structurally related ORFs are marked in red, DNA-or enzyme-related ORFs are marked in green, and hypothetical proteins are marked in gray.

The genome of JNUWH1 (NCBI accession No. OR544901) is 44,051 bp in length, with the GC content of 43.67%. Bioinformatic analysis identified 68 open reading frames (ORFs), with 35 hypothetical proteins of unknown functions (Figure 4A). The genome of JNUWD (NCBI accession No. OR544902) spans 43,278 bp, exhibiting the GC content of 43.87%. The phage genome encompasses 66 predicted ORFs, harboring genes responsible for various biological functions (Figure 4B), with 35 ORFs annotated as hypothetical proteins. The substantial proportion of putative ORFs with no assigned function underscores the importance of future research to uncover the roles of these hypothetical proteins. The two genomes contain many genes encoding proteins involved in phage cleavage cycle, e.g., helicase and ssDNA binding protein, and proteins that may be involved in the bacteriophage resistance to bacteria, e.g., endonuclease, exonuclease, and methyltransferase. PhageTerm analysis reveals these phages’ genome ends have direct terminal repeats. Based on tRNA prediction programs, it was revealed in the JNUWH1 and JNUWD genome, sequences encoding tRNAs with CCT anticodons were identified.

### 3.5 Phylogenetic and comparative genomic analysis

The genomes of bacteriophages JNUWH1 and JNUWD have 97.28% nucleic acid sequence similarity. Notably, 10 highly homologous sequences were identified and anchored, with structural genes, including tail fiber proteins, exhibiting remarkable similarity exceeding 95% (Supplementary Table S2). This comprehensive analysis sheds light on the evolutionary relationship and shared genetic elements between JNUWH1 and JNUWD, offering valuable insights into their biological implications and potential applications. Through vContact2 analysis, both investigated bacteriophages have been identified as members of the viral family *Siphoviridae*, consistent with the results obtained from TEM data. This taxonomic assignment is supported by shared genomic characteristics, conserved gene content, and phylogenetic proximity to known *Siphoviridae* family members (Brussow and Desiere, 2001).

**Figure 5:**
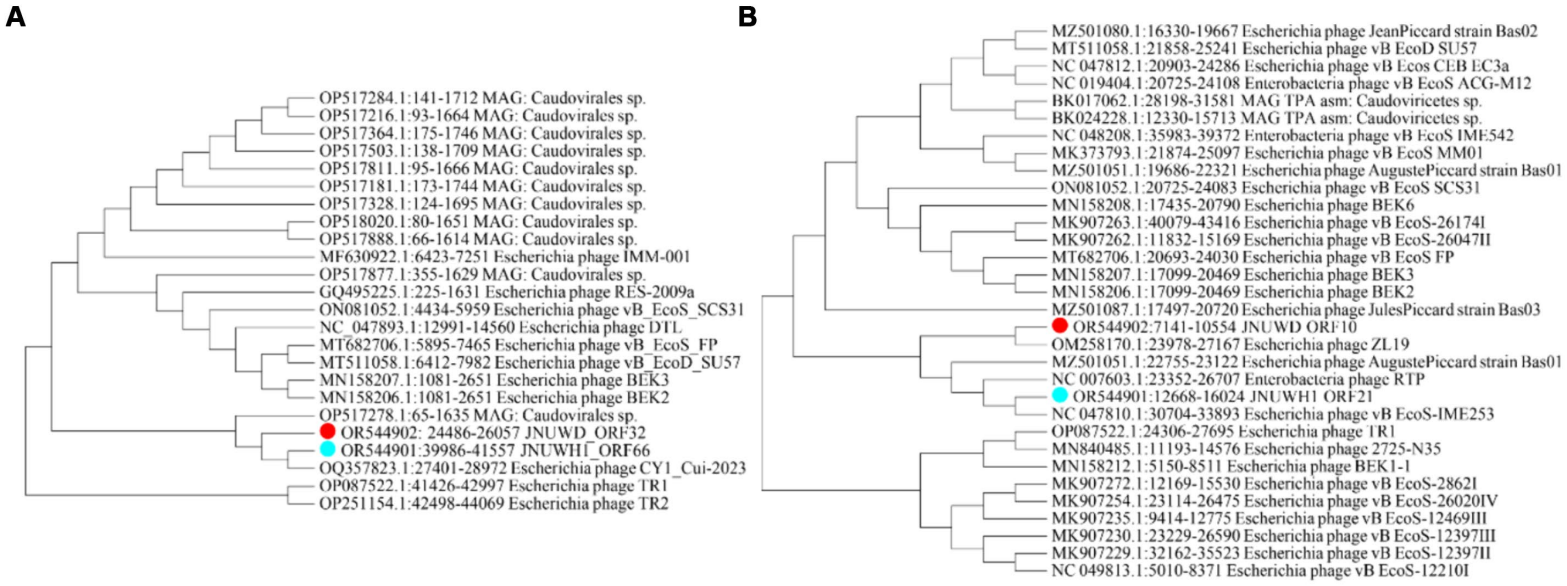
Molecular Phylogenetic analysis by Maximum Likelihood method. **(A)** Phylogenetic tree based on sequence of terminase large subunit. **(B)** Phylogenetic tree based on sequence of tail fiber protein.

The large terminase subunit protein exhibits relatively high conservation among the close relatives of JNUWD1 and JNUWH. For instance, the large terminase protein of *Escherichia* phage CY1_Cui-2023 (accession # OQ357823) shares 99.62 and 99.94% identical DNA sequences with those of JNUWD1 and JNUWH, respectively (Figure 5A). Even when compared to the distantly related large terminase phage proteins in the analysis, which share 93.46% identical residues with phage vB_EcoD_SU57 (Koonjan et al., 2020) (accession # MT511058) in comparison to both JNUWD1 and JNUWH, the level of similarity remains high. The large tail fiber protein, encoded by the genomes of JNUWD and JNUWH1, also shows surprising similarity among the cluster of phages most closely related to JNUWD and JNUWH1, with 89.91% identical DNA sequences. The highest similarity to JNUWD’s tail fiber protein DNA sequence is observed in *Escherichia* phage ZL19 (accession # OM258170.1), reaching 89.26%. Similarly, the highest similarity to JNUWH1’s tail fiber protein DNA sequence is found in *Escherichia* phage vB_EcoS-IME253 (accession # NC_047810.1), with a similarity of 94.07%. However, proteins from the more distantly related group of phages are not particularly similar (Figure 5B). For instance, phage vB_EcoD_SU57’s tail fiber (accession # MT511058) shares only 86.49 and 85.87% identity, with significant differences observed in the C-terminal end. It is noteworthy that the observed differences in tail fiber proteins between JNUWD and JNUWH1 are primarily concentrated at the C-terminal end. Despite their high relatedness at the genomic level, these differences suggest the potential for variations in host cell surface receptor specificity.

### 3.6 Identification and confirmation of phage receptor-related genes

**Figure 6:**
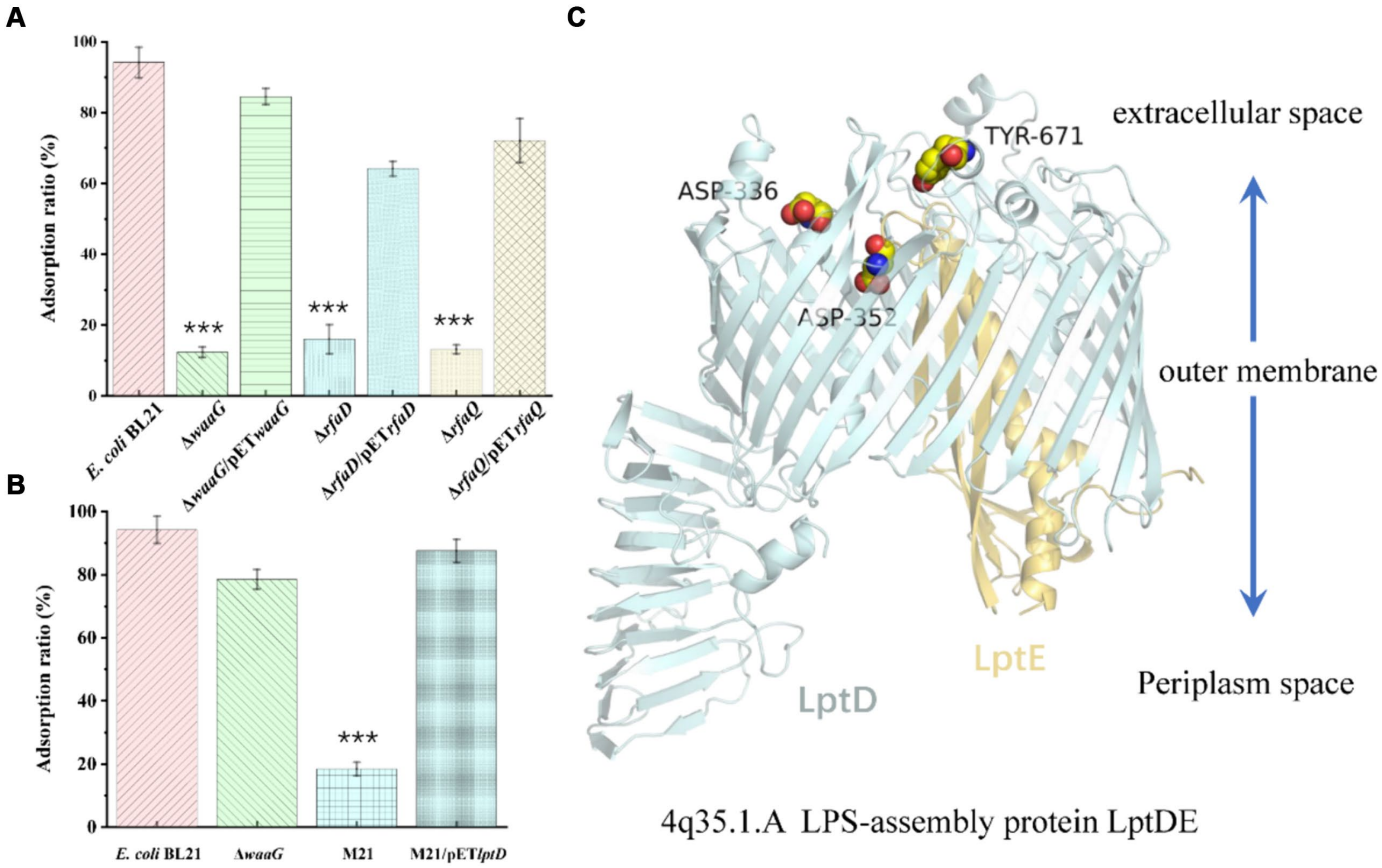
**(A)** Adsorption properties of phage JNUWH1 **(B)** Adsorption properties of phage JNUWD. **(C)** The site of mutation in the lptDE of mutant M21. The error bars indicate standard deviation. ***, *p* < 0.001. The result was calculated based on three repeated experiments.

We sought to replicate natural evolutionary processes through co-cultivation of host *E. coli* and bacteriophages (Farquharson et al., 2021). This method was employed to selectively screen for phage receptor-associated proteins. Following comprehensive genomic resequencing, we established that the mutations in *rfaD*, *waaG*, and *rfaQ* conferred resistance to the JNUWH1 bacteriophage in the host *E. coli*. Notably, the genes *rfaD*, *waaG*, *rfaQ* are integral to lipopolysaccharide (LPS) biosynthesis, transfer, and assembly (Heinrichs et al., 1998; Washizaki et al., 2016). This substantiates that JNUWH1 recognizes LPS as its primary receptor. To further corroborate this finding, we conducted tests assessing the adsorption rate of the *waa*G-deficient, *rfaD*-deficient, and *rfaQ*-deficient strain (*E. coli* BL21 Δ*waa*G, *E. coli* BL21 Δ*rfaD*, *E. coli* BL21 Δ*rfaQ*) against the JNUWH1 bacteriophage. The LPS deficient strain exhibited a resistance to JNUWH1, with an adsorption rate of 12.4–16.1%, in contrast to the wild-type *E. coli* BL21 which demonstrated an adsorption rate of 94.2% (Figure 6A). When the wild type genes in the mutant strain were complemented using pET-28a (pET28a*waaG*, pET28a *rfaD*, pET28a*rfaQ*), it was observed that the strain regained sensitivity to the JNUWH1 bacteriophage. This underscores the inhibitory effect of LPS absence on the adhesion process during the invasion phase of JNUWH1.

Upon evolving *E. coli* to resist the JNUWD bacteriophage, we made an intriguing observation. Mutations in the lptD protein, which is involved in a variety of biological processes, including organic solvent tolerance, hydrophobic antibiotic resistance and membrane permeability (Frisinger et al., 2023), specifically Y671D/D336Y/D352Y, conferred resistance to the phage JNUWD. However, upon utilizing the *waaG-*deficient strain to ascertain whether LPS serves as the receptor for JNUWD, results were negative. Therefore, we hypothesized that the extracellular portion of LPS exposed does not act as a recognition receptor for JNUWD (Nakao et al., 2012; Washizaki et al., 2016). Upon evaluating the adsorption rate of the naturally evolved strain M21, we observed that mutations in the gene *lptD*, pertinent to outer membrane structure (Li et al., 2015; Skurnik et al., 2020), led to an adsorption rate of 18.4% (Figure 6B). We attempted to construct an lptD-deficient strain, however, the deletion of *lptD* led to growth limitations in *E. coli* (Frisinger et al., 2023), rendering successful knockout unattainable. Consequently, Therefore, we expressed the wild-type *lptD* gene by pET28a plasmid in the lab-evolved resistant strain M21. Our research revealed that the complemented strain exhibited restored sensitivity to the bacteriophage JNUWD, indicating a successful recovery of bacteriophage susceptibility. This supports our hypothesis that *lptD* plays a pivotal role as the receptor-associated gene for the JNUWD bacteriophage. According to the reported crystal structure of lptD (Protein Data Bank with the accession code 4Q35)(Qiao et al., 2014), the mutated points (Figure 6C) are positioned in regions of the lptD protein that are exposed to the extracellular space. This finding provides an explanation of protein structure that the mutations at these three points enable M21 to evade recognition by JNUWD.

## 4 Discussion

This study presents a comprehensive exploration of the genomic and biological characteristics of bacteriophages JNUWH1 and JNUWD. Their remarkable 97.28% homology, despite being isolated separately from distinct regions and at different times, raises intriguing questions about the convergence of phage evolution. This suggests a dynamic interplay between these phages and their bacterial hosts, driving the adaptation and evolution of phages in response to evolving bacterial resistance (Qiao et al., 2014). Their robust lytic activity and prolonged inhibition of bacteria hold great promise in clinical contexts (Chevallereau et al., 2021), especially in scenarios where traditional antibiotics face challenges.

Through advanced genomic techniques, we uncovered a high degree of extensive genomic modularity (Smug et al., 2023), exemplified by the identification of highly homologous sequences and the conservation of crucial structural genes. Notably, tail fiber proteins exhibited an exceptional degree of sequence similarity exceeding 95%, underscoring their functional significance in phage-host interactions.

From a taxonomic perspective, both phages were confidently classified within the Siphoviridae family, aligning with their shared genomic characteristics. Additionally, their biological traits, including thermal and pH stability, host range, and one-step growth curves, provide valuable insights into their potential applications in various industrial and clinical contexts. From our findings, it is evident that the isolated bacteriophages have a limited host range. Therefore, according to previous reports, replacing the cauda protein can expand or change the specific host selection, and in view of the potential application of JNUWH and JNUWD in anti-infection, we are inclined to explore ways to expand the host range, and possibly develop super phages with wider clinical applications (Chen et al., 2017).

In conclusion, phage convergence, evidenced by their high degree of homology despite their different origins, reveals the complex evolutionary dynamics of phage immunity to bacteriophage resistance. This study not only advances our understanding of phage biology but also highlights their potential as versatile tools in combating bacterial infections. The genetic flexibility observed at the molecular level opens new avenues for exploring phage-host interactions and their applications in biotechnology and medicine. In situations where conventional antibiotics encounter challenges, particularly in scenarios requiring novel therapeutic approaches, the screening of bacteriophages for clinical utility frequently reveals instances where phages targeting specific antibiotic-resistant bacteria exhibit inadequate inhibitory capabilities. Nonetheless, the robust lytic activity and prolonged inhibition of bacteria observed in phages also indicate their considerable clinical promise. Diversifying the host range of these phages exhibiting potent lytic activity presents a unique strategy in the advancement of clinical antibacterial phage therapy. This discovery lays the foundation for further investigation aimed at maximizing the clinical potential of these remarkable bacteriophages.

## Data availability statement

The datasets presented in this study can be found in online repositories. The names of the repository/repositories and accession number(s) can be found at: https://www.ncbi.nlm.nih.gov/genbank/, No. OR544901 https://www.ncbi.nlm.nih.gov/genbank/, No. OR544902.

## Author contributions

HZ: Conceptualization, Data curation, Formal analysis, Investigation, Methodology, Project administration, Software, Supervision, Validation, Visualization, Writing – original draft, Writing – review & editing. JY: Conceptualization, Formal analysis, Supervision, Validation, Visualization, Writing – review & editing. XP: Conceptualization, Data curation, Formal analysis, Methodology, Resources, Supervision, Writing – review & editing. YH: Data curation, Investigation, Methodology, Visualization, Writing – review & editing. ZZ: Conceptualization, Data curation, Software, Validation, Writing – review & editing. XZ: Conceptualization, Data curation, Formal analysis, Funding acquisition, Investigation, Project administration, Resources, Supervision, Visualization, Writing – review & editing. WZ: Conceptualization, Formal analysis, Project administration, Resources, Visualization, Writing – review & editing. ZR: Conceptualization, Funding acquisition, Project administration, Resources, Supervision, Visualization, Writing – review & editing.

## Footnotes

^1^ https://blast.ncbi.nlm.nih.gov/Blast.cgi
