# Supplementary material for: Genomic and biological insights of bacteriophages JNUWH1 and JNUWD in the arms race against bacterial resistance
Source: Front Microbiol. 2024 Jun 26;15:1407039. doi: 10.3389/fmicb.2024.1407039 (PMC11233448; doi:10.3389/fmicb.2024.1407039)
Supplement: Supplementary file 1 [file Data_Sheet_1.pdf]

Supplementary Material

1 Supplementary Figures and Tables

1.1 Supplementary Tables

Supplementary Table 1

Specific Name	Serotypes	Source of Strains	Strain description
**E. coli**	O157:H7	ATCC 43895	https://www.atcc.org/products/all/43895.aspx
**E. coli**	O55:H7	CB9615	https://doi.org/10.1371/journal.pone.0008700
**E. coli**	O157:H7	ATCC 35150	https://www.atcc.org/products/all/35150.aspx
**E. coli**	O157:H7	ATCC 700728	https://www.atcc.org/products/all/700728.aspx
**E. coli**	O111:K58:H21	ATCC 29552	https://www.atcc.org/products/all/29552.aspx
**E. coli**	O103:H8	ATCC 11229	https://www.atcc.org/products/all/11229.aspx
**E. coli**	O78:K80	CICC 10421	http://www.china-cicc.org/cicc/downpdf/?sid=554
**E. coli**	O78:H11	ATCC 35401	https://www.atcc.org/products/all/35401.aspx
**E. coli**	O128:H7	CDC 1458-80	https://www.atcc.org/products/33638
**E. coli**	O114:H2	ATCC 23540	https://www.atcc.org/products/all/23540.aspx
**E. coli**	O145:H2	BAA-2585	https://www.atcc.org/products/baa-2585-pack
**E. coli**	O26:H11	ATCC BAA-2196	https://www.atcc.org/products/baa-2196dq
**E. coli**		ATCC 10798	https://www.atcc.org/products/all/10798.aspx
**E. coli**		ATCC 70026	https://www.atcc.org/products/700926
**E. coli**	O111:H8	CDC 1997-3215	https://www.atcc.org/products/baa-179

Supplementary Table 2

Start of JNUWH1	End of JNUWH1	Start of JNUWD	End of JNUWD	Length of match in JNUWH1	Length of match in JNUWD	identity (%)
458	1527	21564	20480	1070	1085	86.87
2200	8277	20412	14335	6078	6078	97.62
9227	10143	13390	12474	917	917	85.88
10769	15534	12453	7688	4766	4766	94.02
17524	18809	5932	4647	1286	1286	95.73

20342	21214	2919	2050	873	870	78.73
23336	35415	42205	30126	12080	12080	97.28
36725	38059	28727	27393	1335	1335	97.45
38430	39407	27043	26075	978	969	97.35
39985	44051	26058	21992	4067	4067	99.19

1.2 Supplementary Figure

Supplementary Figure 1

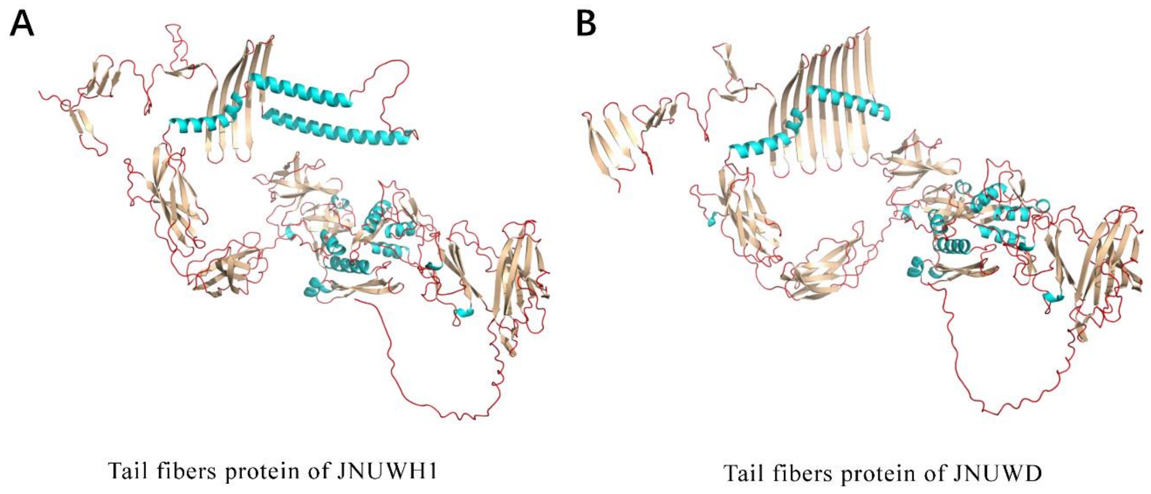

The above two protein structures were modeled by Alphafold2.3 and illustrated by pymol2.0
